# Supplementary material for: COVID-19 Vaccination and Vaccine Hesitancy in the Gaza Strip from a Cross-Sectional Survey in 2023: Prevalence, Risk Factors, and Associations with Health System Interventions
Source: Vaccines (Basel). 2024 Sep 26;12(10):1098. doi: 10.3390/vaccines12101098 (PMC11511228; doi:10.3390/vaccines12101098)
Supplement: Supplementary file 1 [file vaccines-12-01098-s001.zip › vaccines-3176379-supplementary.pdf]

Table S1. Gaza Strip population size estimates (2021)

No	Governorate	Geolocality name	Geolocality type	Population in geolocality (2021)	Number of clusters	Number of households	Code assigned geolocality (survey)	Number of clusters allocated in sample	Number of households selected
1	محافظة شمال غزة North Gaza Governorate	North Gaza Governorate		416906	288	71880			
2		Um A Nasser	Rural	5352	4	923	591	1	7
3		Beit Lahiaبِيت لاهْيا	Urban	101507	70	17501	588	19	110
4		Beit بِيت حَانُون Hanoun	Urban	59022	41	10176			
5		Jabalia مُخَيَّم جَبَالْيا Camp	Camp	55887	39	9636	590	10	60
6		Jabaliaجَبَاليا	Urban	195137	135	33644			
7	محافظة غزة Gaza Governorate	Gaza Governorate		713488	492	123015			
8		Al Shati مُخَيَّم الشَاطِىء Camp	Camp	44535	31	7678	593	5	72
9		Gazaغزّة	Urban	645576	445	111306	594	44	235
10		Al مدينة الزهراء Zahra'a City	Urban	5836	4	1006			
11		Moghraqa Alالمُغْراقَ	Rural	12527	9	2160	592	3	16
12		Joh Al جُحْر الدِيك Deek	Rural	5014	3	864			
13	محافظة الوسطى Middle Governorate	Middle Governorate		302507	209	52156			
14		Al مُخَيَّم النُصَيْرَات Nuseirat Camp	Camp	35153	24	6061			
15		Nuseirat Alالنُصَيْرَات	Urban	60735	42	10472			
16		Al Bureij مُخَيَّم البُرَيْج Camp	Camp	31030	21	5350	598	10	79
17		Bureij Alالبُرَيْج	Urban	17153	12	2957			
18		Zawayda Alالزَوايْدَة	Rural	26398	18	4551			
19		Deir Al مُخَيَّم دِير البَلح Balah Camp	Camp	7734	5	1333			

20		AL مُخَيَّم المَغَازِي Maghazi Camp	Camp	20105	14	3466			
21		Maghazi Alالمَغَازِي	Urban	10707	7	1846			
22		Deir Al دِير البَلَح Balah	Urban	83192	57	14343			
23		Musader Alالمُصَدَّر	Urban	2865	2	494	601	2	16
24		Al Salqa وادِي السَلْقَا Valley	Rural	7435	5	1282	607	2	36
25	محافظة خانيونس Khan Younis Governorate	Khan Younis Governorate		413727	285	71332			
26		Qarara Alالقَرَارة	Urban	32376	22	5582			
27		Khan مخيم خانيونس Younis	Camp	45970	32	7926	614	3	87
28		Khanخانيونس Younis	Urban	228972	158	39478			
29		بَنِي سُهِيلا	Urban	46257	32	7975			
30		عَبَسَان الجَدِيدَة	Urban	10370	7	1788			
31		عَبَسَان الكَبِيرَة	Urban	29879	21	5152	609	2	55
32		خُزَاعَة	Rural	12712	9	2192	615	2	36
33		الفُخَّارِي	Rural	7192	5	1240			
34	محافظة رفح Rafah Governorate	Rafah Governorate		260117	179	44848			
35		Rafahرَفَح	Urban	191185	132	32963	618	13	79
36		Rafah مُخَيَّم رَفَح Camp	Camp	40651	28	7009	619	3	20
37		Al Nasrالنصر	Urban	9992	7	1723			
38		Shoka Alالشُوكَة	Rural	18290	13	3153	616	2	13

Table S2. Comparison of vaccination and vaccine hesitancy between 2021 and 2023

	2021 survey		2023 survey		
**Characteristic**	**N = 906**[1]	**95% CI**[2]	**N = 894**[1]	**95% CI**[2]	**p-value**
**Received Vaccine**	49.1%	43.1%, 55.1%	63.5%	59.4%, 67.5%	<0.001
**Vaccine Hesitant**	34.1%	28.1%, 40.6%	31.7%	27.8%, 35.6%	0.381

[1]% Weighted %
[2]CI = Confidence Interval

Table S3. Respondents' self-reported vaccination history

Variable	Frequency (N=572) [1]	%[2] (95% CI)[3]
**Manufacturer**		
Pfizer only	335	61.7 (56-67)
Sputnik only	160	24.2 (20-29)
Pfizer/Sputnik	3	0.5 (0.15-1.8)
Pfizer and other	7	1.4 (0.59-3.4)
Other combinations	67	12.1 (9.5-15)
**Number of doses**		
One	240	43.5 (38-49)
Two	290	48.6 (43-54)
Three	40	7.9 (5.4-11)
**First dose received**		
2020	58	7.6 (5.1-11)
2021	340	55.8 (50-61)
2022	174	36.6 (32-42)
**Last dose received**		
2020	38	6.2 (3.8-9.9)
2021	315	50.6 (45-56)
2022	218	42.9 (38-48)
2023	1	0.2 (0.03-1.6)

[1] Excludes vaccinations where the manufacturer was unknown (n=7) and vaccinations with suspected date errors where the date of first or last vaccination was prior to 2020 (n=4) and where the date of first vaccination was reported to be after the date of last vaccination (n=12).
[2] Weighted percentage
[3] CI = Confidence Interval

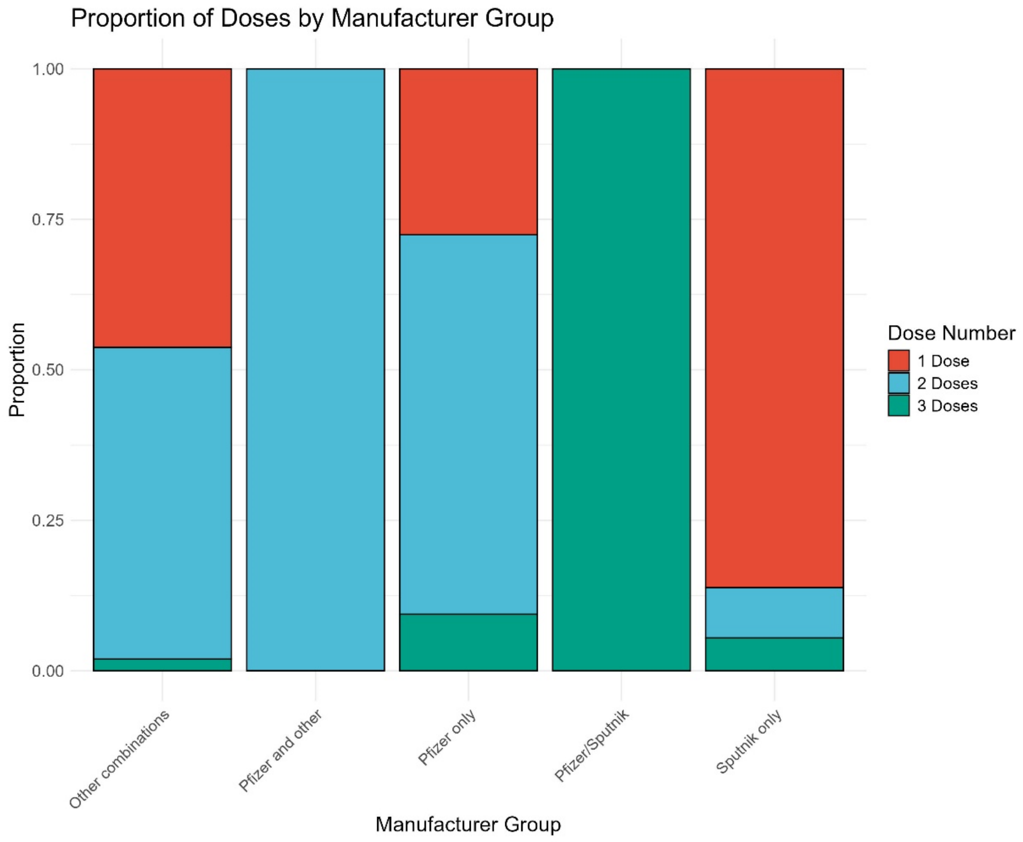

Figure S1. Proportion of doses of COVID-19 vaccine received by manufacturer
